# Supplementary material for: Investigating the Dynamic Variation of Skin Microbiota and Metabolites in Bats During Hibernation
Source: Biology (Basel). 2025 Nov 23;14(12):1648. doi: 10.3390/biology14121648 (PMC12729892; doi:10.3390/biology14121648)
Supplement: Supplementary file 1 [file biology-14-01648-s001.zip › Supplementary material.pdf]

Investigating the dynamic variation of skin microbiota and metabolites in bats during hibernation

Fan Wang[1], Wendi Song[1], Denghui Wang[1], Zihao Huang[1], Mingqi Shan[1], Shaopeng Sun[1], Zhouyu Jin[1], Jiaqi Lu[1], Yantong Ji[1], Keping Sun[2], Zhongle Li[1,3,*]

[1] College of Life Science, Jilin Agricultural University, Changchun 130118, China.

[2] Jilin Provincial Key Laboratory of Animal Resource Conservation and Utilization, Northeast Normal University, Changchun 130117, China.

[3] Jilin Provincial International Cooperation Key Laboratory for Biological Control of Agricultural Pests, Changchun 130118, China.

* Corresponding authors: Zhongle Li, lizhongle@jlau.edu.cn

Table S1. Sampling time points and detailed information.

Date	Hibernation period	Type	Site	Sample size	Temperature (mean±SD)	BMI[a] (mean±SD)
10.23,2023	Early	Temporal	Dalazi cave	10	6.58±1.57	0.38±0.05
2.25,2024	Middle	Temporal	Dalazi cave	10	6.39±0.46	0.34±0.02
4.4,2024	Late	Temporal	Dalazi cave	9	8.32±0.40	0.31±0.02
4.5,2024	Late	Spatial	Dalazi cave	10	7.38±0.85	0.32±0.02
4.5,2024	Late	Spatial	Temple cave<br>Gezi cave	10	8.97±0.72	0.30±0.34

a: BMI, Body mass index.

Table S2. The microbial sources of metabolites were identified by MetOrigin2.0 software.

Table S3. Detailed information of the reagents.

Regent	CAS	Item Number	Brand
Melatonin	73-31-4	M821128-5ml	Macklin, Shanghai
Saccharin	81-07-2	S426096-1ml	Aladdin, Shanghai
Neohesperidin	13241-33-3	N408193-1ml	Aladdin, Shanghai
Prostaglandin E2	363-24-6	P793307-1ml	Macklin, Shanghai
L-Glutathione oxidized	27025-41-8	T88217-1ml	Yuanye, Shanghai
(5-L-Glutamyl)-L-Amino Acid	5875-41-2	T26999-5mg	Yuanye, Shanghai
L-Glutamic acid	56-86-0	R22703-100ml	Yuanye, Shanghai
Astaxanthin	472-61-7	A794364-1ml	Macklin, Shanghai
L-arginine	74-79-3	V32817-1ml	Yuanye, Shanghai

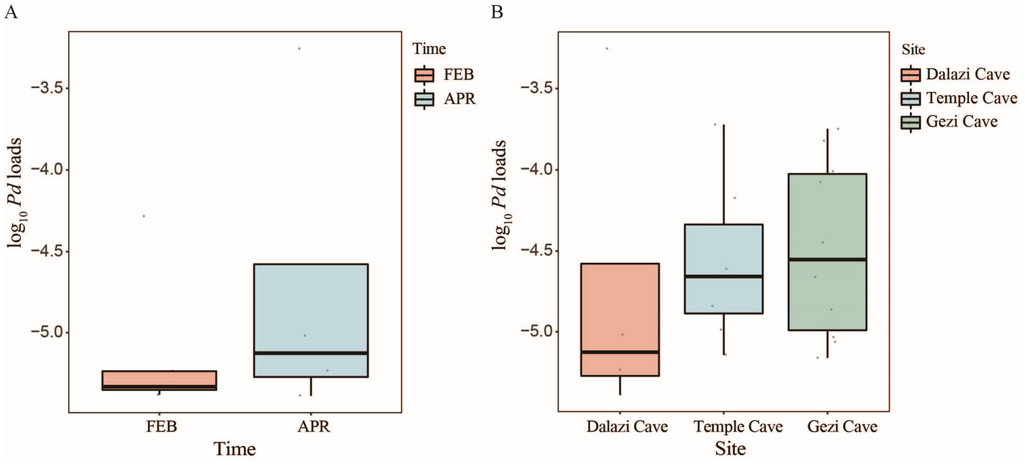

Figure S1. Analysis of *Pd* infection in bats during hibernation. (A) *Pd* loads in bats at different time points. (B) *Pd* loads in bats from different locations.

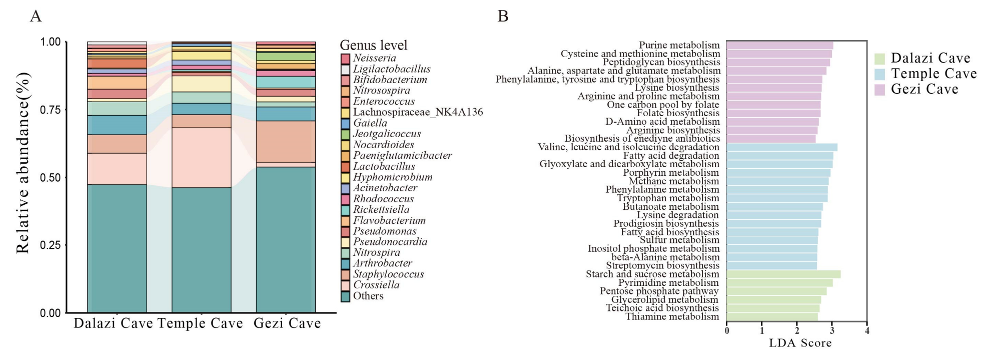

Figure S2. Composition and functional prediction of bat skin bacterial communities across different locations. (A) Stacked bar plot of major bat skin bacteria at the genus level with relative abundance greater than 1%. (B) KEGG pathways of bat skin bacteria based on Linear Discriminant Analysis (LDA score > 2.5).

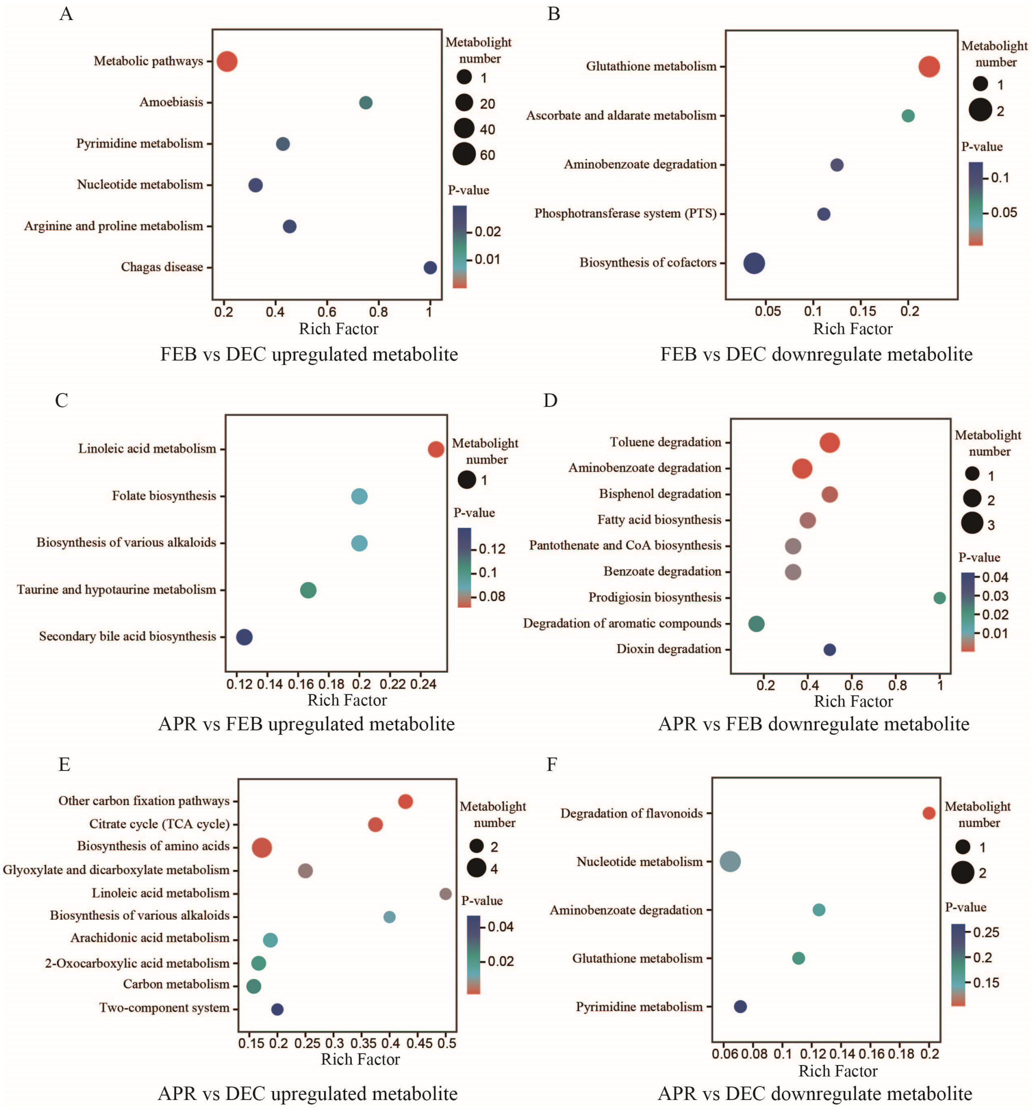

Figure S3. KEGG pathway enrichment of differentially regulated metabolites across

hibernation stages. Pathways significantly upregulated ($P < 0.05$) from early to middle hibernation (A) and the top five downregulated pathways (B). Top five upregulated pathways from middle to late hibernation (C) and pathways significantly downregulated ($P < 0.05$) (D). Pathways significantly upregulated ($P < 0.05$) from early to late hibernation (E) and the top five downregulated pathways (F).

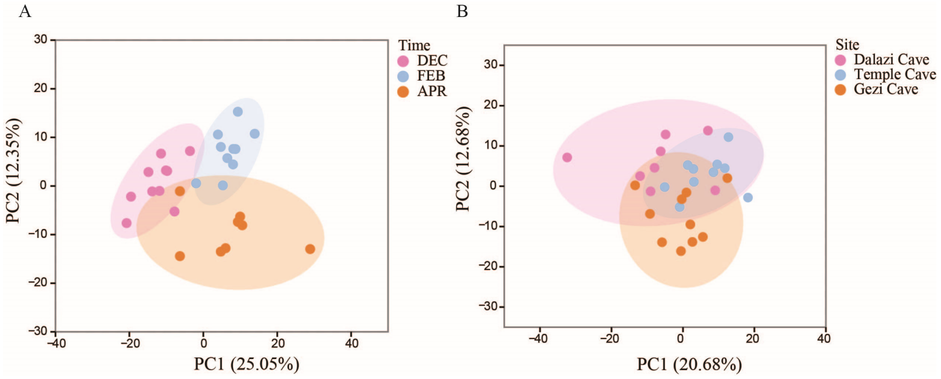

Figure S4. Multivariate statistical analysis of bat skin metabolites across different temporal and spatial scales. Principal component analysis during hibernation (A) and across hibernation locations (B).
